# Metamizole Utilization and Expenditure During 6-Year Period: Serbia vs. Croatia

**DOI:** 10.3389/fpubh.2018.00213

**Published:** 2018-07-26

**Authors:** Milijana Miljkovic, Viktorija Dragojevic-Simic, Nemanja Rancic, Radoje Simic, Tanja Pekez-Pavlisko, Aleksandra Kovacevic, Dusica Stamenkovic

**Affiliations:** ^1^Centre for Clinical Pharmacology, Medical Faculty Military Medical Academy, University of Defence, Belgrade, Serbia; ^2^Department for Plastic Surgery, Institute for Mother and Child Health Care of Serbia “Dr. Vukan Cupic”, Medical School, University of Belgrade, Belgrade, Serbia; ^3^Family Medicine Clinic “Tanja Pekez-Pavlisko”, Kutina, Croatia; ^4^Clinic for Anesthesiology and Critical Care, Medical Faculty Military Medical Academy, University of Defence, Belgrade, Serbia

**Keywords:** metamizole, utilization, expenditure, adverse drug reactions, Serbia, Croatia

## Abstract

**Background:** Metamizole is a medication with analgesic, antipyretic, spasmolytic, and weak anti-inflammatory effects. The aim of our study was to evaluate a six-year trend in the utilization and expenditure of metamizole in comparison to other group of licensed non-opioid analgesics in Serbia and Croatia, in order to rationalize its use and prescribing in these countries.

**Methods:** The data of metamizole vs. all other non-opioid analgesics utilization and expenditure in Serbia and Croatia was analyzed according to the WHO methodology and expressed as defined daily doses per 1,000 inhabitants per day (DDD/1,000 inhabitants/per day) and total costs, respectively, during the 6-year period from 2010 to 2015.

**Results:** In the observed period, utilization of metamizole was 3.31 fold higher in Serbia than in Croatia (median in Serbia was 2.238 vs. 0.675 in Croatia DDD/1,000 inhabitants/per day/per year). Expenditure of metamizole in the same period was 5.29-fold higher in Serbia than in Croatia (median in Serbia was 1,738,192.51 €/per year vs. 328,355.03 €/per year in Croatia).

**Conclusion:** Utilization and expenditure of non-opioid analgesics, including metamizole, in Serbia was significantly higher comparing with Croatia.Further research is needed to determine whether the current analgesic consumption in Serbia meets the needs of the patient. The benefits of metamizole should be weighed against the risk of metamizole-induced adverse effects. Until then, its prescribing should be based on indications and the appropriate duration of therapy.

## Introduction

Metamizole (noramidopyrine-methane-sulfonate, dipyron) is a nonsteroidal anti-inflammatory drug (NSAID), with analgesic, antipyretic, spasmolytic, and weak anti-inflammatory properties. Based on its chemical structure it belongs to the group of pyrazolones, along with phenazone, aminophenazone, propyphenazone, nifenazone (1, 2). Metamizole spasmolytic effect is unique among NSAIDs. Indications for its use are postoperative pain, acute injury, cancer and colic pain, and migraine (1, 3–5).

Metamizole was synthesized in 1920, and introduced into the clinical practice under brand name Novalgin® in Germany in 1922 (1, 6). Central and peripheral mechanisms are responsible for its actions (3), but the exact mechanism of its analgesic effect remains partially unclear. The peripheral antinociceptive effect of metamizole is mediated by inhibition of cyclooxygenase (COX) enzyme mediated prostaglandins biosynthesis, predominantly COX enzyme type 2 (1). The COX-2 inhibition is responsible for its antipyretic effect (7, 8). Analgesic effect are explained with its action on blocking COX-3 isoform and the impact on the opioidergic and cannabinoid system (9, 10). As naloxone blocks the analgesic effect of metamizole, interaction with the endogenous opioid system is proposed as one of the possible mechanisms for its analgesic properties (8). Moreover, interactions with the endogenous peroxidase, cannabinoid, and glutamate systems are under consideration (11).

The review of the therapeutic benefits and risks of adverse drug reactions (ADRs) should always be assessed in order to achieve the proper balance between therapeutic efficacy and safety risks.

Metamizole use was associated with abdominal discomfort, maculopapular rash, Stevens-Johnson syndrome, Lyell syndrome, agranulocytosis, hypotension, bronchospasm, arrhythmia and allergic reactions (12). Agranulocytosis, thrombocytopenia, anemia, aplastic and haemolytic anemia, and leucopenia significantly limited its utilization in everyday practice (1).

Agranulocytosis, is rare, but life threatening ADR. Risk for metamizole-induced agranulocytosis is estimated as 0.06 and 1.1 cases per million users per week of treatment (13). Agranulocytosis is defined as a neutrophil count of less than 5 × 10^9^ L^−1^ (<500 μL^−1^) (14). The risk for agranulocytosis is increasing with prolonged application of metamizole, but after the 10th day from the last dose of metamizole, the risks for agranulocytosis is declinig (1). The clinical signs and symptoms of agranulocytosis can appear from several days to up to 3 months after introducing metamizole into therapy (15). Metamizol caused agranulocytosis can be explained by immunological and toxic reaction. Immune-mediated agranulocytosis mechanism suggests that the drug or its metabolite irreversible bind to the neutrophiles membrane, and T lymphocytes or antibodies are causing cell lysis subsequently (7, 16). In the toxic reactions, the drug directly damages the myeloid granulocyte precursor cells (7). Agranulocytosis is predominantly hypersensitive type reaction and high dosage metamizole exposure over prolonged period of time increases the probability for sensitization and later development of metamizole-related agranulocytosis (17). Several factors may affect agranulocytosis-induced mortality, such as the length of metamizole use, general condition of the patient, the use of antibiotics and the growth stimulation factors for granulocytic precursors in the treatment of agranulocytosis (18). According to some authors, metamizole therapy for inpatients has low possibility to cause significant sensitization, mostly due to its short term of application and medical supervision (14). On the contrary, as OTC drug, metamizole utilization is not under proper supervision.

It is important to note that the first bone marrow transplantation in the former Yugoslavia was performed in the patient with agranulocytosis as ADR of metamizole. Among other analgesics that could cause agranulocytosis are indomethacin, oxyfenbutazone, and phenylbutazone. All adverse reactions to pyrazolones are not dose-dependent (19).

Therefore, there is a different policy regarding metamizole usage among countries. In many of them, including United States of America, United Kingdom and Japan, it is withdrawn from the market; in some of them, it is available only as a prescription drug for strictly defined indications, while in Mexico, Brazil and China it can be obtained as an OTC drug (6, 7, 20).

Data concerning metamizole consumption and expenditure are scarce and not readily available. According to ATC/DDD classification system, metamizole utilization should be analyzed within the group N02B, “other analgesics and antipyretics” (21, 22).

The aim of this study was to analyze and evaluate a six-year trend in utilization and expenditure of metamizole in Serbia, in relation to other registered non-opioid analgesics, and to compare those parameters in Croatia.

## Materials and methods

We performed retrospective, observational, cross-sectional study. The data concerning metamizole consumption during a 6-year period (from 2010 to 2015) in Serbia and Croatia were retrieved from editions of the publication “Marketing and consumption of medicinal products for use in human medicine,” issued on yearly bases by the “Medicines and Medical Devices Agency of Serbia” (ALIMS) (https://www.alims.gov.rs/eng/) and the Agency for Medicinal Products and Medical Devices of Croatia (HALMED) (http://www.halmed.hr/en/O-HALMED-u/). The data were compared between those two countries as the Croatia is one of the countries considered referral for our own, by the Republic Health Insurance Fund (23).

The data on the metamizole and other groups of non-opioid analgesics utilization was analyzed according to the Anatomical Therapeutic Chemical/Defined Daily Doses (ATC/DDD) methodology (2, 13, 24). Results were expressed as the number of defined daily doses per 1,000 inhabitants per day (DDD/1000 inhabitants/day) (25, 26).

Results were calculated and presented for the following groups of non-opioid analgesics, according to the ATC codes: M01AB, Acetic acid derivatives and related substances (diclofenac, acemetacin, ketorolac, indometacin, lonazolac, aceklofenac); M01AC, Oxicams (meloxicam, lornoxicam, tenoxicam); M01AE, Propionic acid derivatives (ibuprofen, ketoprofen, naproxen, flurbiprofen, tiaprofenic acid, dexketoprofen); M01AH, Coxibs (etoricoxib, celecoxib); M01AX, Other antiinflammatory and antirheumatic agents, non-steroids (glucosamine, nimesulide, oxaceprol); N02BA, Salicylic acid and derivatives (acetylsalicylic acid); N02BB, Pyrazolones (metamizole sodium, fenazon); and N02BE, Anilides (paracetamol).

The median (IQR- interquartile range: range between 25th and 75th percentile) of DDD/1,000 inhabitants/day for metamizole and other non-opioid analgesics in the above mentioned six-year period was calculated, as well as total expenditure (median and IQR) of observed drugs for both countries. Expenditure was calculated and expressed in euros (€) (based on National Bank of Serbia average annual course RSD-€ (http://www.nbs.rs/internet/english/scripts/ kl_prosecni.html) and Croatian National Bank average annual course HRK-€ (https://www.hnb.hr/en).

The statistical analysis was performed using “Microsoft Office Excel 2007” software. Data from both countries in observed six-year period were used for trend analysis. The continual variables were presented as median (IQR).

## Results

**Figure 1:**
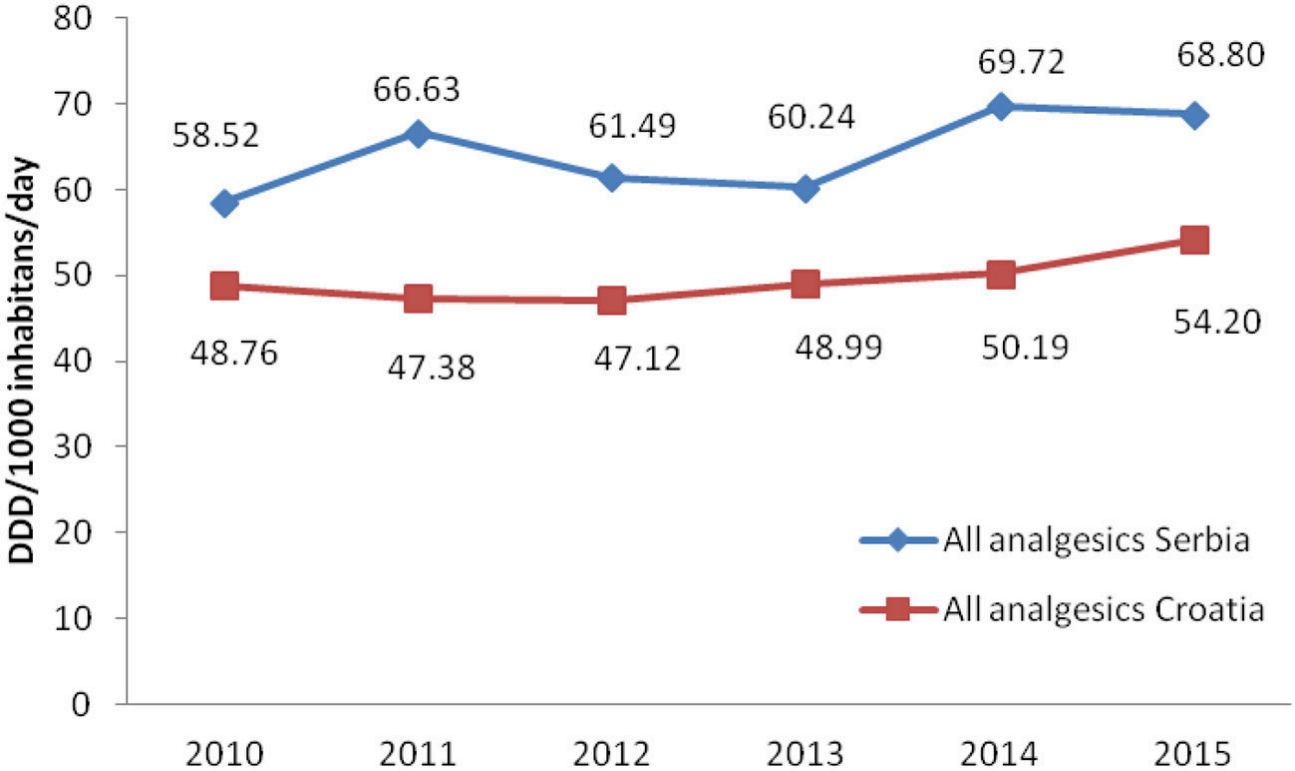
Utilization of all non-opioid analgesics in Serbia and Croatia (period 2010–2015) (DDD/1,000 inhabitans/day).

**Figure 2:**
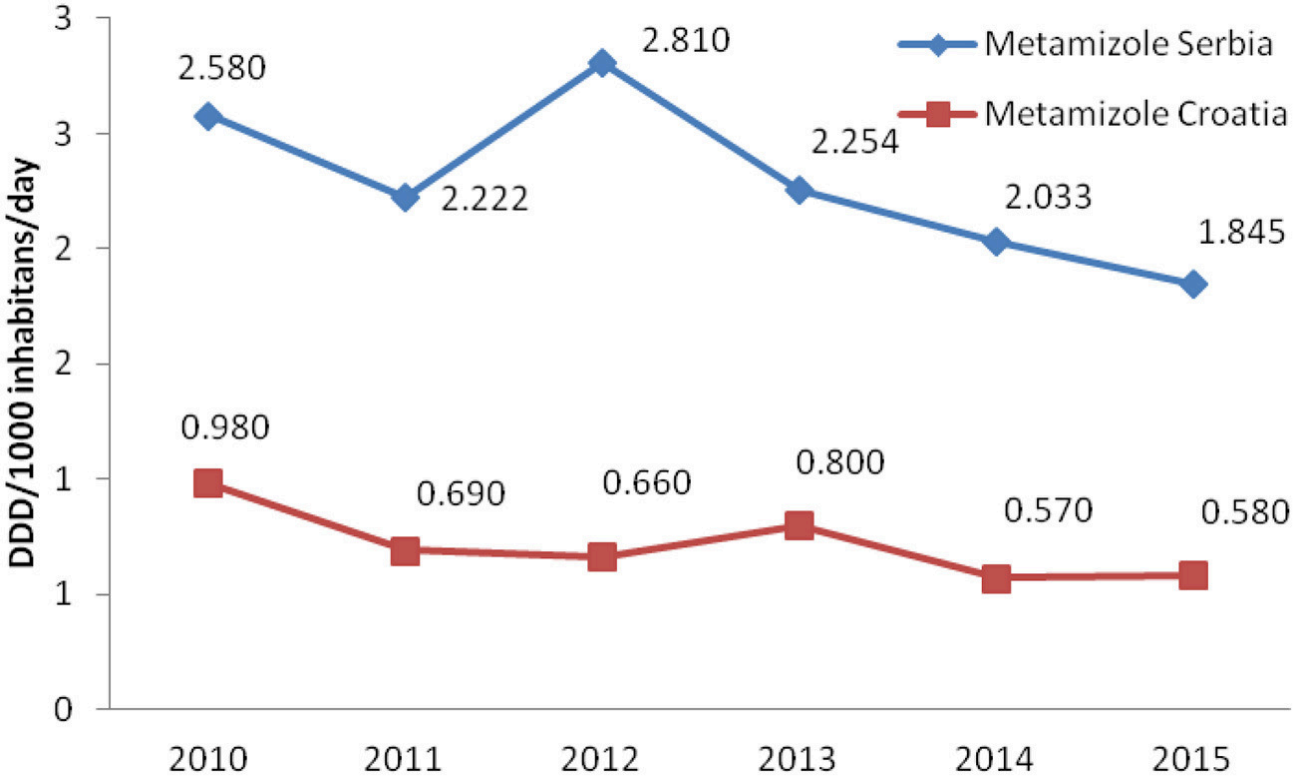
Utilization of metamizole in Serbia and Croatia (period 2010–2015) (DDD/1,000 inhabitans/day).

**Figure 3:**
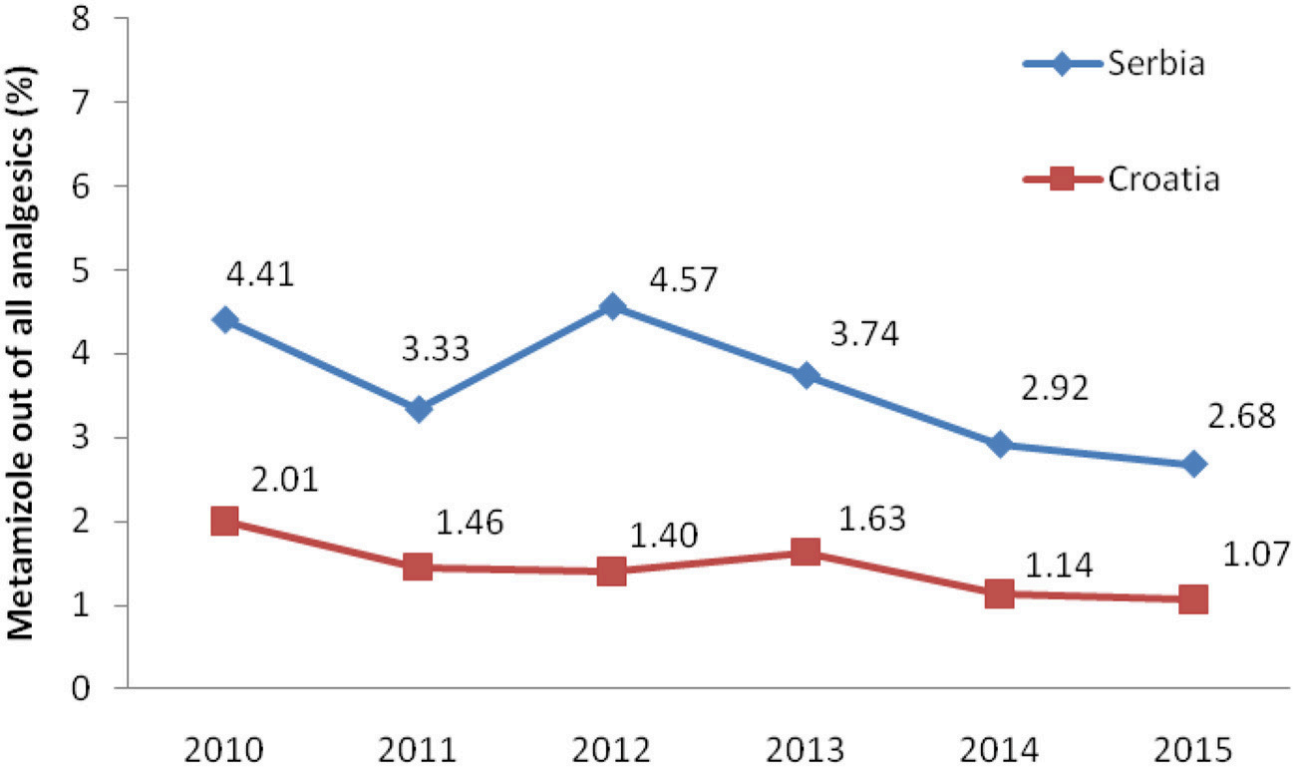
The proportion of metamizole utilization out of all non-opioid analgesic in Serbia and Croatia (DDD/1,000 inhabitans/day).

Total non-opioid analgesics consumption was higher in Serbia compared to Croatia in the observed six-year period (2010-2015) expressed as DDD/1,000 inhabitans/day (Figure 1). In each analyzed year, metamizole consumption was higher in Serbia than in Croatia (Figure 2). The proportion of metamizole utilization (DDD/1,000 inhabitans/day) out of all non-opioid analgesic in Serbia and Croatia had decreasing trend (Figure 3).

Based on the obtained data, the utilization of metamizole was 3.31 times higher in Serbia than in Croatia, i.e., median for six-year period in Serbia was 2.238 DDD/1,000 inhabitants/per day/per year vs. 0.675 in Croatia.

**Figure 4:**
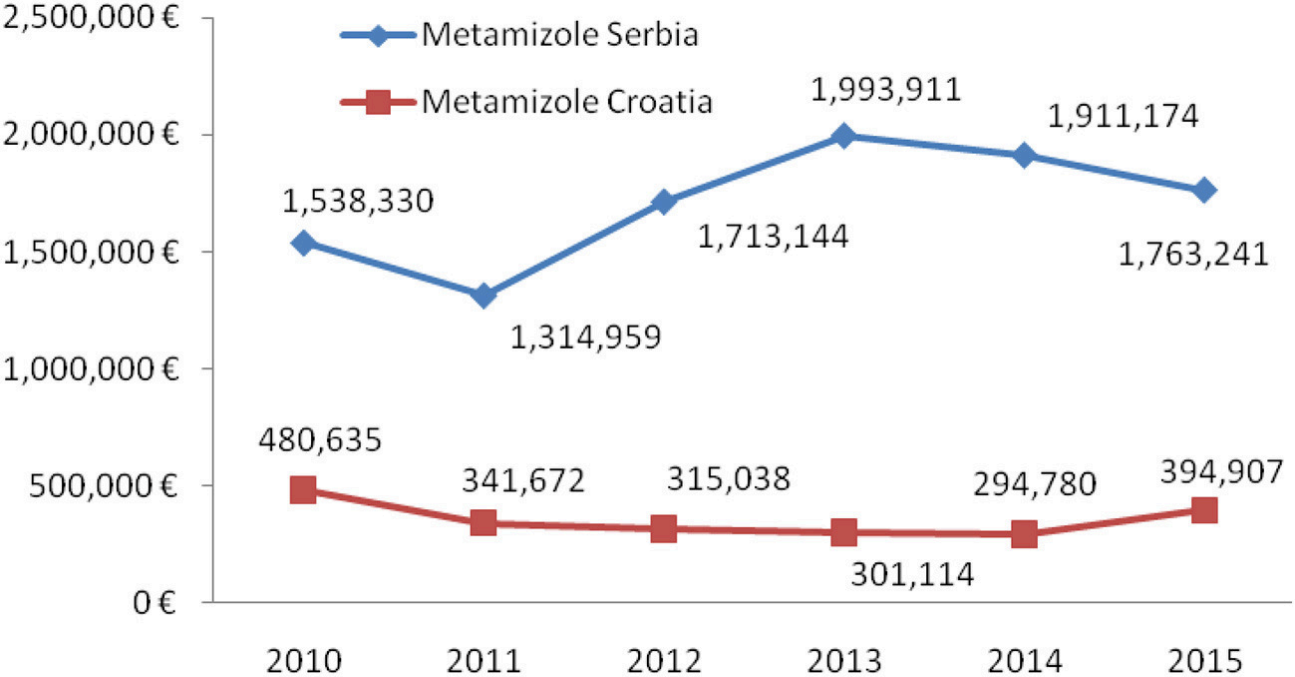
Expenditure of metamizole in Serbia and Croatia (period 2010–2015).

**Figure 5:**
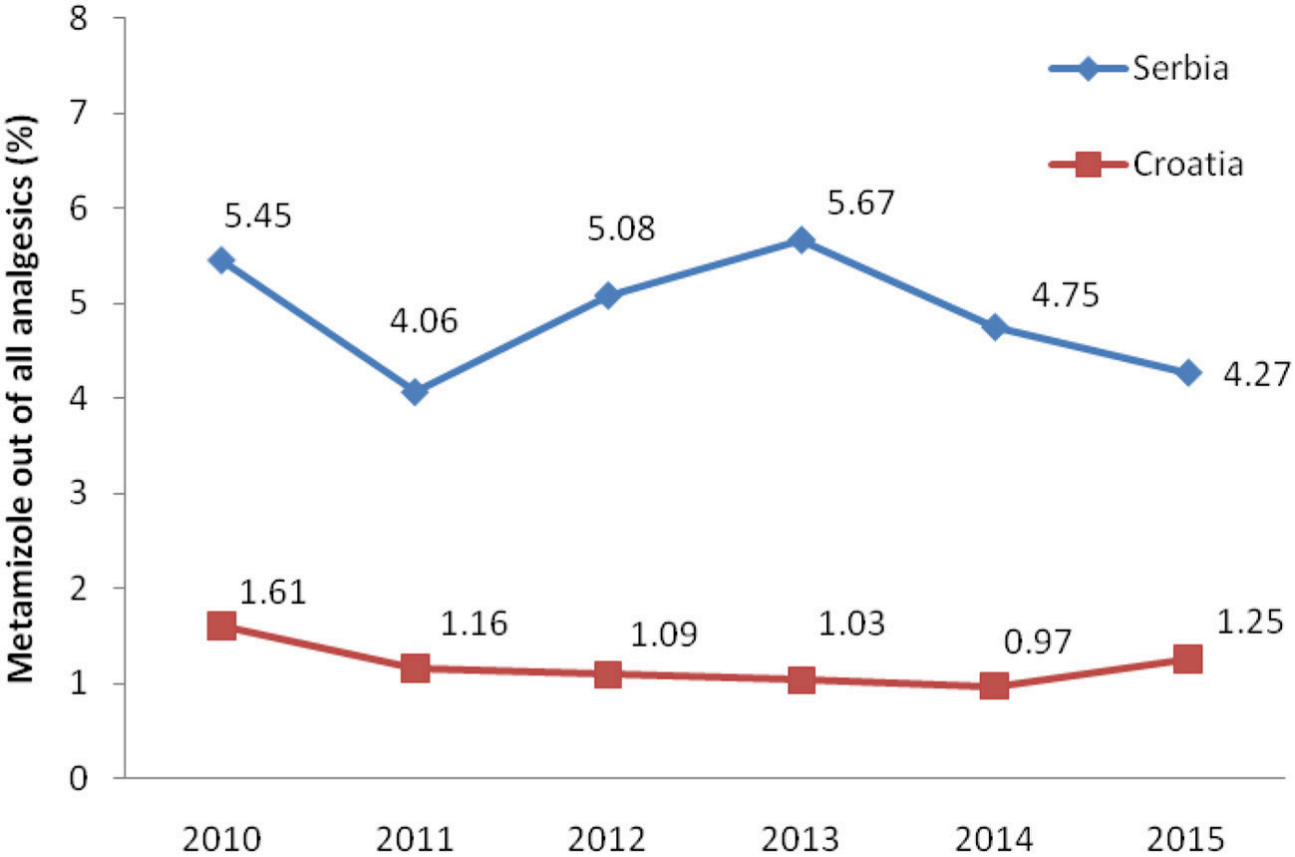
The proportion of metamizole expenditure out of all non-opioid analgesic in Serbia and Croatia (period 2010–2015).

Total expenditure for all non-opioid analgesics was higher in Serbia comparing with Croatia in the observed six-year period (Table 1). The expenditures for all subcategories of analgesic for both countries are presented in Table 1. The costs for metamizole have been rising, as a whole, in Serbia, while in Croatia a decreasing trend was observed (Figure 4). However, the proportion of metamizole expenditure out of all non-opioid analgesics in both countries was declining (Figure 5). Data presented in Table 2 show a rising trend of costs when nominal values of metamizole expenditure (€/per inhabitant/year) are calculated.

**Table 1:** Expenditure^*^ of non-opioid analgesic drugs in Serbia and Croatia (period 2010–2015).

**ATC code**	**Country**	**2010**	**2011**	**2012**	**2013**	**2014**	**2015**	**Median**	**Interquartile range**
M01AB	Serbia	6,565,609.68	11,123,323.26	10,159,939.29	9,249,514.60	11,023,741.17	10,530,415.29	10,345,177.29	8,578,538.37–11,048,636.69
	Croatia	5,528,175.10	4,719,175.83	4,730,554.63	4,421,292.48	4,619,747.71	3,621,631.80	4,669,461.77	4,221,377.31–4,929,959.75
M01AC	Serbia	1,933,066.06	2,822,400.16	1,648,146.17	1,454,259.35	539,995.49	1,567,557.26	1,607,851.71	1,225,693.38–2,155,399.58
	Croatia	1,402,903.11	1,231,144.75	1,022,446.82	865,535.88	735,170.38	702,786.85	943,991.35	727,074.50–1,274,084.34
M01AE	Serbia	7,247,607.07	5,735,827.64	4,415,600.36	8,316,324.86	9,197,363.97	10,406,946.36	7,781,965.96	5,405,770.82–9,499,759.57
	Croatia	8,106,301.21	8,675,882.73	9,157,244.04	10,011,528.36	11,215,691.48	12,124,900.13	9,584,386.20	8,533,487.35–11,442,993.64
M01AH	Serbia	9,403.14	131,330.92	220,965.98	324,417.30	270,522.69	305,102.75	245,744.33	100,848.97–309,931.39
	Croatia	0.00	2,365.73	1,975.52	1,521.11	59,387.81	259,680.55	2,170.62	1,140.83–109,460.99
M01AX	Serbia	2,253,440.02	3,196,439.24	2,985,773.11	3,159,383.44	3,087,448.77	2,904,568.77	3,036,610.94	2,741,786.58–3,168,647.39
	Croatia	0.00	0.00	0.00	0.00	340.89	1,989.48	0.00	0.00–753.04
N02BA	Serbia	2,287,323.33	2,988,274.31	3,451,666.65	3,194,735.56	3,641,094.53	3,338,477.83	3,266,606.69	2,813,036.56–3,499,023.62
	Croatia	3,484,688.90	3,350,549.13	2,303,432.53	2,359,047.76	2,283,220.97	2,392,281.07	2,375,664.41	2,298,379.64–3,384,084.07
N02BB	Serbia	1,538,329.69	1,314,959.28	1,713,143.69	1,993,910.53	1,911,174.48	1,763,241.33	1,738,192.51	1,482,487.09–1,931,858.49
	Croatia	480,634.64	341,672.38	315,037.69	301,113.59	294,780.21	394,907.09	328,355.03	299,530.24–416,338.98
N02BE	Serbia	6,365,794.20	5,065,884.46	9,117,520.58	7,471,883.11	10,575,952.06	10,515,882.33	8,294,701.84	6,040,816.76–10,530,899.76
	Croatia	10,879,807.44	11,155,305.31	11,406,703.17	11,255,035.49	11,203,496.72	11,975,514.06	11,229,266.10	11,086,430.84–11,548,905.89
Total	Serbia	28,200,573.19	32,378,439.27	33,712,755.83	35,164,428.75	40,247,293.16	41,332,191.92	34,438,592.29	31,333,972.75–40,518,517.85
	Croatia	29,882,510.40	29,476,095.86	28,937,394.40	29,215,074.67	30,411,836.17	31,473,691.03	29,679,303.13	29,145,654.60–30,677,299.88

* *Costs are expressed in euros (€)*.

**Table 2:** Expenditure^*^ of metamizole vs. all non-opioid analgesics in Serbia and Croatia per inhabitant/year for period 2010–2015.

	**Year**	**2010**	**2011**	**2012**	**2013**	**2014**	**2015**	**Average**
Serbia (€/per inhabitant/year)	Metamizole	0.21	0.18	0.24	0.28	0.27	0.25	0.24
	Non-opioid analgesics	3.92	4.51	4.69	4.89	5.60	5.75	4.89
Croatia (€/per inhabitant/year)	Metamizole	0.11	0.08	0.07	0.07	0.07	0.09	0.08
	Non-opioid analgesics	2.54	2.61	2.67	2.63	2.62	2.80	2.64

* *Costs are expressed in euros (€)*.

Analysis of data obtained from national data basis pointed out that Serbian health care system spent almost 5.3 times more money for metamizole in comparison to Croatia during the observed period (median for six-year period in Serbia was 1,738,192.51 €/per year vs. 328,355.03 in Croatia).

## Discussion

The results presented in this study showed higher levels of all non-opioid analgesics consumption in Serbia than in Croatia. In Croatia, in 2007–2013, the ATC N02B group was the only one of the four analyzed groups of NSAIDs that showed an overall decrease in total consumption (21).

The analysis of total consumption of metamizole in Serbia in the period from 2010 to 2015 based on the annual issues of the publication “Marketing and consumption of medicinal products for use in human medicine” published by ALIMS, expressed as DDD/1,000 inhabitants per day, indicates a gradual, but constant decline in metamizole consumption in the observed six-year period.

Tends observed in Lithuania in the period from 2005 to 2007, showed 8.46% increase of medicines consumption which belongs to the ATC N02B group. Simultaneously, metamizole consumption decreased (22).

In Serbia, metamizole is available as metamizole sodium formulations designed for oral, intravenous and intramuscular administration, registered under the trade names Novalgetol® and Analgin®. It is registered for short-term use in severe posttraumatic and postoperative pain, when therapy with other non-opioid analgesics is unsuccessful (3). Therapeutic regimen for the treatment of severe pain includes maximum therapy duration of 72 h, with a single dose of 2.5 g b.i.d. The other dosing regimen includes a single dose of 1 g q.8h, with a flexibility to titrate dose in order to obtain satisfactory analgesic or antipyretic response, with maximum recommended daily dose of 4 g (3, 27). Metamizole is registered in Croatia under a trade name Alkagin®, solution for injection 2.5 g/5ml, and Analgin® tablets in a dose of 500 mg for treatment of short-lasting severe pain of different origin (postoperative, posttraumatic, cancer pain, abdominal pain as colic, and pain in other acute and chronic conditions), if other therapeutic interventions are insufficient (28). Metamizole is also available as antipyretic, when high body temperature persists in spite of other applied medical interventions.

In both countries (Serbia and Croatia) paracetamol was the most prescribed analgesic and its consumption was increasing from 2010 to 2015, except in 2015 in Serbia, when an insignificant decrease in consumption was observed. The most likely reason for high utilization of paracetamol could be its favorable benefit/risk ratio at an acceptable price, which makes it one of the most common prescribed antipyretic and analgesic in children. According to the results from study conducted in Finland, as a representative country with high standards of pain therapy and pharmacovigilance as well, analgesics and antipyretics, including NSAIDs (ATC groups N02, M01), were the most common used medications among OTC medicines in children (29).

The lowest consumption within analgesic groups in Serbia was registered for coxibs. The possible limitations for prescribing this group of NSAIDs are narrow indications and higher prices in relation to other analgesics, whereas in Croatia increased consumption of coxibs was obeserved in the last two years.

A constant decline in consumption of acetylsalicylic acid is evident in the both countries. Lower dose of this medication needed for the prevention of cardiovascular diseases than dose for analgesic treatment could explain such trend.

Drugs within the ATC group M01AB (acetic acid derivatives i.e., diclofenac, ketorolac, indomethacin) were more utilized in Serbia for pain treatment than medications within the M01AE group (propionic acid derivatives), regardless of the fact that ibuprofen is considered to be the safest conventional NSAID concerning the gastrointestinal side effects (30). In Croatia, propionic acid derivatives were utilized more than in Serbia. M01AE is the only analgesic group which was utilized more frequently in Croatia than in Serbia. In a study conducted in 15 countries worldwide, including Australia, People's Republic of China, Malaysia, Taiwan, Canada, and the UK, diclofenac was the most commonly utilized anti-inflammatory drug, along with ibuprofen, naproxen, and mefenamic acid (31). Regarding NSAIDs adverse effects such as gastrointestinal toxicity, ketoprofen, piroxicam and azapropazon have the highest potential for such events, while diclofenac and ibuprofen expressed relatively low risk for such ADRs (30). However, the problem remains, as the most used NSAIDs (ibuprofen, diclofenac, naproxen) are available as OTC medications with increasing trends in consumption.

The total expenditure for non-opioid analgesics increased over a 6 year period both in Serbia and in Croatia. Highest costs were observed for paracetamol during the whole period.

The higher consumption of metamizole in Serbia was accompanied with the higher expenditure, since it was found that 5.3 times more money was spent on this drug in comparison to Croatia. Moreover, the expenditure per inhabitant per year for metamizole in Serbia was 2.89 times higher than in Croatia (32, 33).

Krnic et al. analyzed consumption of opioid and nonopioid analgesics in Croatia during period from 2007 to 2013 (21). The N02B group was the only group, among four analyzed, that showed an overall decrease in total consumption, during examined seven-year period. Consumption of metamizole expressed as DDD/1,000 inhabitants/day was continually decreasing from year 2007 to 2012, and its continuous decrease in sale and expenditure throughout the whole seven-year period was present (21). In Serbia, analysis of total consumption of metamizole in the period from 2010 to 2015, also indicated gradual decline in metamizole consumption (16, 17), but overall costs for metamizole were slightly increasing from year 2011, onwards. Costs of metamizol amounted a share of 4.85% of the total costs for all analyzed drugs in Serbia, whereas it was 1.19% in Croatia.

The trend of reduced utilization of metamizole could be explained by two reasons. Better quality of monitoring and registering of adverse reactions to metamizole resulted in increased number of adverse reaction reports, based on WHO VigiAccess database records and influenced medical practitioner's decision to opt for another analgesics. On the other hand, other non-opioid medications, like paracetamol and COX-2 selective inhibitors, were gaining more popularity, based on more favorable characteristics, including safety, or at least they appeared like safer drugs.

The most common reported adverse reactions to metamizole, both according to WHO data, as well as ALIMS data, were the skin and subcutaneous tissue disorders. The constant increase in the number of reported adverse reactions to metamizole has been noticed since 2007.

Moreover, skin reactions, like pemfigus vulgaris and mild forms of skin rash, including urticaria, were also recorded in VigiAccess base (20). Rarely, metamizole can cause generalized drug reactions (4). Life threatening reactions, including Stevens-Johnson's syndrome, toxic epidermal necrolysis, and anaphylaxis, are rare. In WHO UMC VigiAccess base in a period from 1968 to 2018, 236 cases (0.78%) of Stevens-Johnson syndrome and 199 cases (0.65%) of toxic epidermal necrolysis were reported (4, 20). Although anaphylactic reaction could occur after enteral routes of administration, it is more frequent after parenteral metamizole administration (3, 34). Reported gastrointestinal disorders caused by metamizole include nausea, vomiting, abdominal pain, diarrhea, upper abdominal pain etc. (20). These adverse effects have been described only when high doses of oral and parenteral metamizole administration (35). Metamizole has mild adverse event profile on the upper gastrointestinal tract, compared to other NSAIDs and its administration is safer in patients with an increased risk of gastrointestinal bleeding or renal disease compared to other NSAIDs (36).

Moreover, extensive epidemiological investigations showed that short-term exposure to metamizole does not increase risk of agranulocytosis and it is lower than risk for aplastic anemia connected with application of indometacine or diclofenac (16).

The number of reported ADRs regarding medicines belonging to the ATC groups M and N in Croatia is not very large. In 2010, ketoprofen for topical administration was the only one of all NSAIDs with a safety issue, mostly due to observed rare but serious photosensitivity adverse reactions (37). In 2015, there were documented several ADR reports regarding NSAIDs, causing mostly nausea, gastrointestinal intolerability, hepatotoxicity, major and minor bleeding. The only emphasissed safety issue in the same report was related to the cases of uptake of ibuprofen high doses, for more than 2,400 mg daily, for a long time that led to increased risk for cardiovascular events (38).

Metamizole has become more popular in Europe in last 10 years, as soon as an epidemiological study showed that the risk for adverse reactions to metamizole was similar to paracetamol and even lower than to acetyl salicylic acid (5).

In spite of its utilization for more than 90 years, the efficacy of metamizole have neither been well documented nor there were enough large randomized clinical trials to show its benefits comparing with other NSAIDs.

## Conclusion

Consumption of all non-opioid drugs in Serbia was significantly higher than in Croatia. However, consumption of metamizole was decreasing in both countries in the observed period. Overall costs of metamizole in Croatia were steadily decreasing, except in the last observed year, 2015. In Serbia, overall costs of metamizole were slightly increasing and amounted a share of 4.85% of the total costs for all analyzed drugs in Serbia.

The benefits of metamizole should be evaluated in the context of metamizole-induced agranulocytosis. For short-term use in the hospital settings, metamizole seems to be a safe choice when compared to other analgesics. However, its place in the contemporary pharmacotherapy needs to be reevaluated in the controlled, randomized clinical trials, both concerning efficacy and safety.

## Author contributions

NR, DS, and VD-S jointly designed the study and defined research questions. RN and MM did most of the data mining and extraction, purification of files for missing data and artifacts and statistical analysis. MM, TP-P, VD-S, DS, and RS contributed to the tables and figures creation and interpretation of data. MM, NR, AK, and VD-S drafted the working version manuscript but all authors contributed to the final version to the extent of important intellectual content.

### Conflict of interest statement

The authors declare that the research was conducted in the absence of any commercial or financial relationships that could be construed as a potential conflict of interest.
